# Case Report: A rare case of multiple gastrointestinal autoimmune disorders mimicking autoimmune polyglandular syndrome type 3B

**DOI:** 10.3389/fimmu.2026.1770109

**Published:** 2026-08-11

**Authors:** Zhongmian Zhang, Yuting Xie, Tianqi Zhang, Xiang Li, Aling Wang, Zhenyu Wu, Ruyi Cai, Shengsheng Zhang

**Affiliations:** 1 Digestive Disease Center, Beijing Hospital of Traditional Chinese Medicine, Capital Medical University, Beijing, China; 2 Department of Nephropathy, Beijing Hospital of Traditional Chinese Medicine, Capital Medical University, Beijing, China; 3 Department of Pathology, Beijing Hospital of Traditional Chinese Medicine, Capital Medical University, Beijing, China

**Keywords:** autoimmune gastritis, autoimmune hepatitis, autoimmune polyglandular syndrome type 3B, case report, primary sclerosing cholangitis, ulcerative colitis

## Abstract

Autoimmune polyglandular syndrome type 3 (APS-3) encompasses autoimmune thyroid disease and other autoimmune disorders. APS-3B involving gastrointestinal autoimmune diseases. In this case, due to the coexistence of multiple autoimmune diseases including ulcerative colitis (UC), autoimmune hepatitis (AIH), primary sclerosing cholangitis (PSC), and hypothyroidism after papillary thyroid carcinoma surgery and iron deficiency anemia, we diagnosed autoimmune gastritis (AIG) through endoscopic manifestations, histological and serological tests. After the identification of this disease subtype, the correlations among these diseases within the group strengthened the rationale for implementing targeted screening strategies in these various autoimmune disorders. Future research is expected to identify the targets and new directions for treating these immune-related diseases through these correlational studies.

## Case description

**Figure 1:**
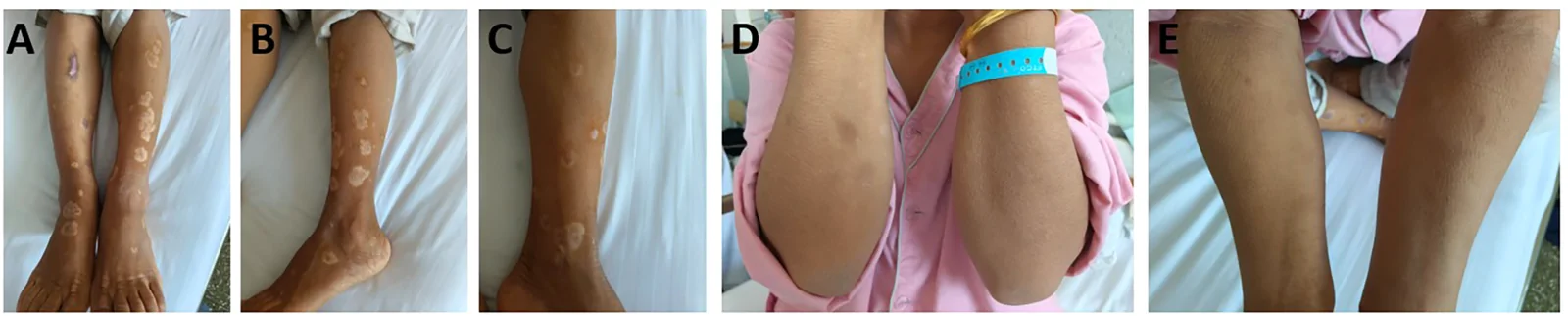
lichen amyloidosis. **(A–E)** Skin manifestations of both lower extremities. Skin manifestations of both upper limbs.

A 40 year-old Chinese woman underwent upper gastrointestinal endoscopy for a reexamination at our hospital. She had a history of ulcerative colitis (UC), autoimmune hepatitis (AIH), primary sclerosing cholangitis (PSC), and underwent total thyroidectomy for papillary thyroid carcinoma, resulting in hypothyroidism. She also presented with lichen amyloidosis (**Figure 1**), and iron deficiency anemia (IDA). The IDA was detected in 2025-05–07 with a hemoglobin of 89 g/L, MCV 64.2 fL, and ferritin of 7.33ng/ml. The patient was initially treated with oral ferrous succinate (400 mg elemental iron daily) for 1week, which resulted in a gradual improvement in hemoglobin to 98 g/L at 1-week follow-up. A comprehensive work-up revealed a transferrin saturation of 2% and serum iron of 2.4 µmol/L. Serum vitamin B12 (608 pg/mL, reference: 197–771 pg/mL) and folic acid (4.61 ng/mL) were within normal ranges, respectively. Intrinsic factor antibody testing was not performed due to assay unavailability. Fecal occult blood tests were negative on multiple occasions. Given that the UC was in clinical and endoscopic remission at the time of IDA evaluation, active gastrointestinal bleeding from UC was considered unlikely. Furthermore, the patient had no history of menorrhagia and a gynecological evaluation was unremarkable. Therefore, the IDA was attributed to impaired iron absorption in the setting of autoimmune atrophic gastritis. Serological markers for autoimmune thyroiditis (TPOAb and TgAb) were not available for retrospective analysis, and the surgical pathology report did not comment on lymphocytic infiltration. Therefore, a definitive diagnosis of autoimmune thyroiditis could not be established. She had multiple autoimmune diseases (AIG, UC, AIH, PSC), which prompted us to consider the possibility of Autoimmune Polyglandular Syndrome Type 3B (APS-3B), although the thyroid component of the syndrome remained unconfirmed. She had multiple autoimmune disease, and long-term mesalazine, Ademetionine, Ursodeoxycholic (UDCA), Euthyrox use.

**Figure 2:**
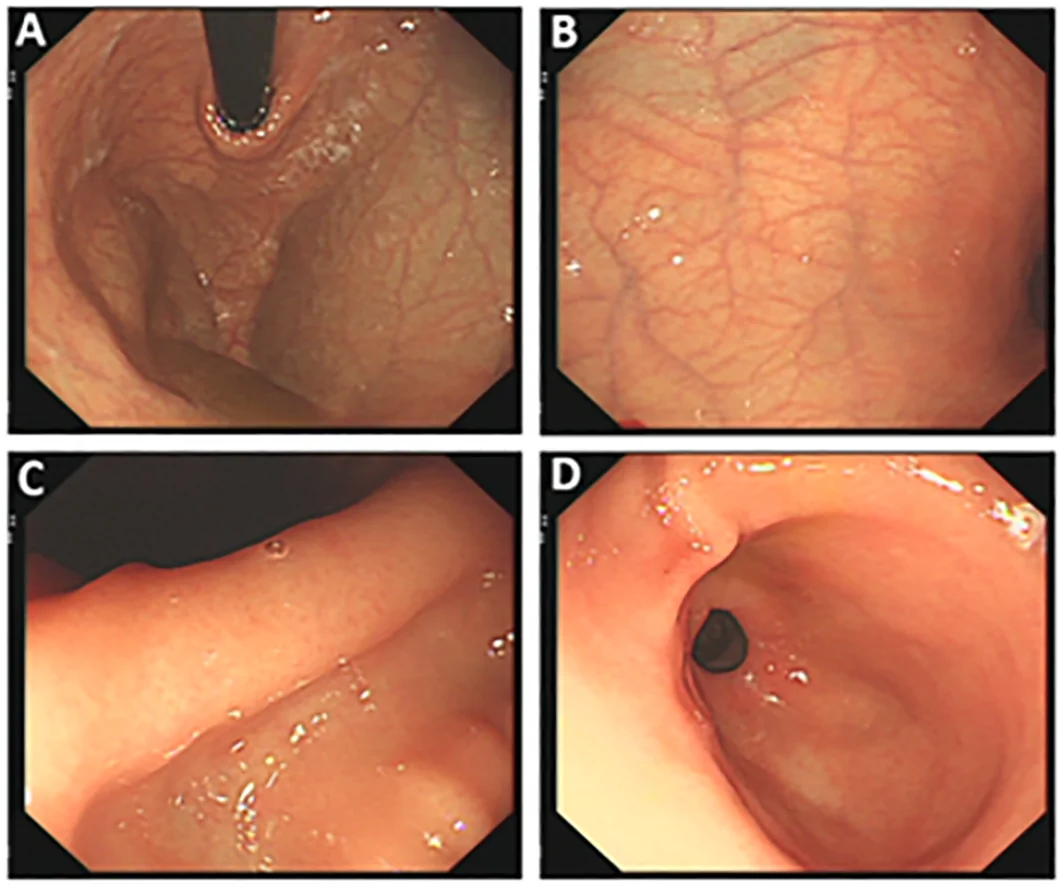
**(A, B)** Grid-like microvessels in the fundus of stomach and greater curvature. **(C, D)** Normal gastric angle and antral mucosa.

Endoscopic examination revealed a normal gastric angle and antral mucosa (**Figures 2C, D**) and a smooth mucosa of the greater curvature of the corpus with a decreasing fold, while reverse atrophy was found in an inflated state. After full inflation, severe mucosal atrophy in the gastric corpus, with grid-like microvessels in the greater curvature of the corpus and fundus of stomach. (**Figures 2A, B**). The Clinical Timeline and Diagnostic Summary of Autoimmune Comorbidities are shown in **Table 1**.

**Table 1:** Clinical Timeline and Diagnostic Summary of Autoimmune Comorbidities.

Disease	Year of onset/diagnosis	Key diagnostic evidence	Treatment/management
Ulcerative Colitis (UC)	2019	Colonoscopy: Continuous mucosal inflammation, vascular pattern loss, erosions from rectum to terminal ileum. Histology: Active chronic colitis with crypt abscesses.	Mesalazine 3g/day (maintenance). Currently in clinical remission.
Autoimmune Hepatitis (AIH)	2016	The liver biopsy in the external hospital provided a clear pathological diagnosis, but the specific report was not available.	—
Primary Sclerosing Cholangitis (PSC)	2016	Labs: Cholestatic pattern (ALP, GGT elevated). MRCP: Multifocal strictures and beading of intrahepatic and extrahepatic bile ducts.	Ursodeoxycholic acid (UDCA) 15mg/kg/day. No dominant strictures requiring endoscopic intervention to date.
Hypothyroidism after Papillary Thyroid Carcinoma surgery	2021	Histopathology: Papillary thyroid carcinoma, no extrathyroidal extension.	Thyroidectomy in 2021. Levothyroxine replacement therapy (Euthyrox 100µg/day).
Iron Deficiency (IDA)	2025	Labs: Hb 89 g/L, MCV 64.2 fL, Ferritin 7.33 ng/ml. Transferrin Saturation 2%.	
Autoimmune Gastritis (AIG)	2021	Endoscopy: Reverse atrophy, grid-like microvessels. Histology: Oxyntic mucosa atrophy, pseudopyloric metaplasia, ECL hyperplasia. Serology: Gastrin 60.36 pmol/L, PGI 18.40 µg/L, PGR 1.24, PCA positive (1:100).	Diagnosed at current presentation. Initiated on oral iron supplements; commenced B12 and folate monitoring. Endoscopic surveillance plan established.

### Hematoxylin-eosin

**Figure 3:**
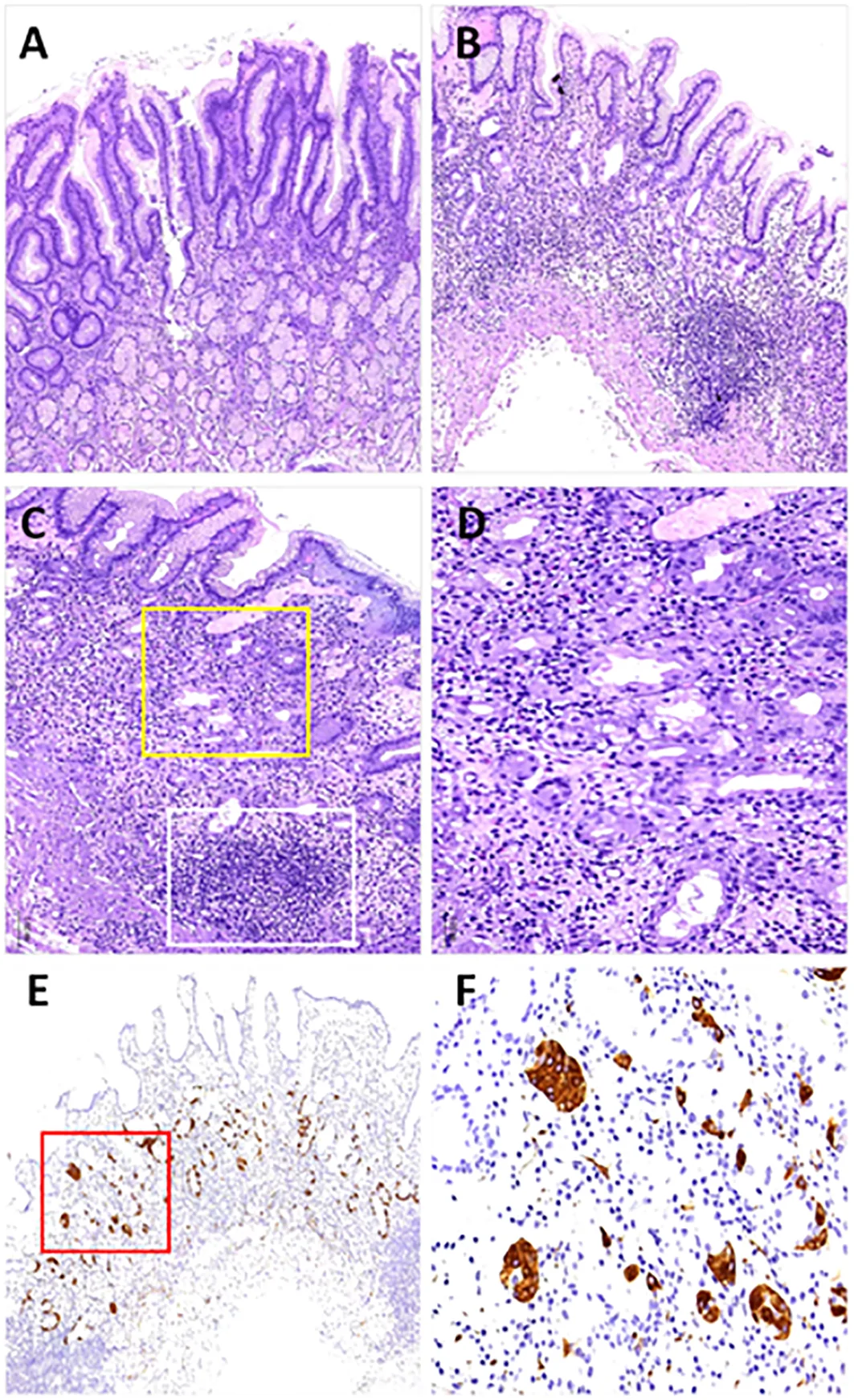
Pathology results. Hematoxylin-eosin (HE) staining results of the gastric antrum **(A)** and corpus **(B)**. **(C)** Tertiary lymphoid structures (TLS) is composed of lymphocytes, and can be seen in **(C)** white frame. Parietal cells show show pseudo-hypertrophy, swelling and degeneration in **(D)** yellow frame and the magnified image is shown in **(D)**. Chromogranin A (Cga) staining revealed linear and tiny nodular hyperplasia of enterochromaffin-like (ECL) cells **(E)**. **(F)** is image of **(E)** enlarged at different magnifications.

Gastric biopsies were obtained from two site: one from the antrum (**Figure 3A**), one from the corpus of greater curvature). The hematoxylin-eosin (HE) staining results of the gastric glands are shown in (**Figure 3**). No significant atrophy of the gastric antrum glands was observed (**Figure 2D**). The degree of chronic inflammation in the deep layer of the gastric fundic mucosa has further intensified, presenting with full-thickness mucosa inflammation, and the degree of inflammation in the deep mucosal glandular areas is more severe than that in the superficial layer (**Figure 3B**). The infiltrating inflammatory cells are mainly lymphocytes, and tertiary lymphoid structures (TLS) can be seen in **Figure 3C** white frame. The gastric fundic glands mucosal layer becomes thinner, and the lamina propria are significantly reduced, presenting atrophic changes mainly characterized by metaplasia of pseudopyloric glands. The hierarchical structure of the fundic glands has basically disappeared (**Figure 3C**), the number of parietal cells has significantly decreased, and the remaining parietal cells show pseudo-hypertrophy, swelling and degeneration (**Figure 3C** yellow frame and **Figure 3D**). Chromogranin A (CgA) staining revealed linear and tiny nodular hyperplasia of enterochromaffin-like (ECL) cells in the acid-secreting mucosal glands of the stomach (**Figures 3E, F**).

### Serological test

Additional blood tests showed a markedly elevated fasting gastrin level of 60.36 pmol/L (reference range: ≤ 5.70 pmol/L). Pepsinogen I (PG I) was severely decreased at 18.40 µg/L (reference: >70.00 µg/L), and pepsinogen II (PG II) was 14.78 µg/L (reference: <11.00 µg/L), resulting in a very low PGI/PGII ratio (PGR) of 1.24 (reference: >7.00). This serological profile (low PGI and low PGR) is a sensitive biomarker for severe corpus-predominant gastric atrophy. The patient was positive for anti-parietal cell antibodies (PCA) at a titer of 1:100. Anti-H. pylori antibody testing was negative, but this serological result does not definitively exclude current or past infection. No histological evidence of H. pylori was found on gastric biopsies, and a rapid urease test was not performed. (**Table 2**)

**Table 2:** Results of the investigations.

Laboratory investigations	Result
Haemoglobin (g/L) (115-150)	89
Mean corpuscular volume (fl) (82-100)	64.2
reticulocyte count% (0.5-2.28)	4.77
Albumin (g/L) (40-55)	38.3
Aspartate aminotransferase (U/L) (13-35)	41.3
Alanine aminotransferase (U/L) (7-40)	40.7
glutamyl transpeptidase (U/L) (7-45)	144.7
Alkaline phosphatase (U/L) (20-150)	212.8
Thyroid-stimulating hormone (mIU/L) (0.56-5.91)	1.390
Free thyroxine (ng/dl) (0.59-1.25)	0.76
Anti-TPO antibody (IU/ml) (0-34)	26.10
Anti-thyroglobulin antibody (IU/ml) (0-115)	16.40
serum iron (μmol/L) (9-27)	2.4
Ferritin (ng/ml) (13-150)	7.33
Transferrin Saturation% (20-55)	2
Gastrin (pmol/l) (≦5.7)	60.36
pepsinogen I (μg/L) (>70)	18.40
pepsinogen II (μg/L) (<11)	14.78
PGR (>7)	1.24
Anti-parietal cell antibody	100
Anti-H.pylori antibody	–
Vitamin B12 (pg/ml) (197-771)	f608
Folic acid (ng/ml) (4.3-19.8)	4.61
lgG(g/L) (7-16)	13.53
lgA(g/L) (0.7-4)	3.17
lgM(g/L)(0.4-2.3)	2

AIG is diagnosed based on the following features: (1) endoscopy: reverse atrophy; grid-like microvessels in the greater curvature of the corpus after full inflation. (2) histopathology: There is a significant infiltration of lymphocytes in the gastric body glands, and TLS can be observed. The mucosal layer becomes thinner, and the intrinsic glands are significantly reduced, presenting atrophic changes mainly characterized by pseudo-pyloric gland metaplasia. The parietal cells show pseudo-hypertrophy, swelling and degeneration. CGA and Syn staining show that the ECL cells on the gastric acid-secreting mucosal glands exhibit linear and nodular hyperplasia. (3) serology: parietal cell antibody (PCA) positivity; PGR 1.24; and (4) medical history: UC, PSC, AIH, hypothyroidism resulting from the resection of papillary thyroid carcinoma, IDA.

## Discussion

This case indicates that when a patient has multiple autoimmune diseases, especially those involving the gastrointestinal tract, the possibility of autoimmune polyglandular syndrome (APS) should be considered. However, a critical distinction must be made: APS-3 requires autoimmune thyroid disease (AIT) as a core component. In our patient, while hypothyroidism was present, it was a consequence of surgical resection for papillary thyroid carcinoma, and we lacked histopathological or serological (TPOAb/TgAb) evidence of concurrent AIT. Therefore, while the clustering of AIG, UC, AIH, and PSC is highly suggestive of APS-3B, we cannot definitively classify this case as such without confirmatory evidence of autoimmune thyroiditis. This limitation underscores the importance of comprehensive autoantibody screening in similar cases.

We suspected autoimmune gastritis because of unexplained IDAs. Just as we had expected, this patient was eventually confirmed as having AIG through both histological and serological tests. Autoimmune destruction of parietal cells leads to hypo/achlorhydria, which impairs the reduction of dietary ferric iron (Fe^3+^) to the absorbable ferrous form (Fe^2+^), thereby reducing duodenal iron uptake (1). This aligns with previous findings reporting a similarly high prevalence of histologically confirmed AIG in autoimmune liver disease (AILD) patients (2). Moreover, since the classification of APS-3 subtypes is based on disease group (3, 4), it can help narrow down the differential diagnosis within specific groups of autoimmune diseases. APS-3B includes AIT and AIG, inflammatory bowel disease, AIH, or primary biliary cholangitis, all gastrointestinal diseases. In this case, the diagnosis of APS-3B was made when PSC, AIH, UC, AIG and hypothyroidism were diagnosed. These associations reinforce the rationale for implementing targeted screening strategies in those multiple autoimmune diseases.

In this case, the presence of multiple autoimmune diseases concurrently with IDA and skin lichenoid changes provided us with clues, which eventually led to the diagnosis of AIG. However, causality and disease progression patterns remain unclear. Long term follow up are necessary to concern gastric neoplasms or the progression of liver pathology. The potential for AIG-related complications like gastric neuroendocrine tumors and atrophic progression to manifest after many years, as shown by long-term data, highlights the critical need for prospective follow-up (5).

Ultimately, it is unknown whether standard AILD immunotherapies (e.g., corticosteroids, azathioprine) protect against, have no effect on, or exacerbate AIG. Clarifying this is crucial for developing integrated management strategies in patients with overlapping autoimmune phenotypes.

## Follow-up and outcome

A multidisciplinary approach involving gastroenterology and hepatology was established. The patient was educated about the increased long-term risk of gastric neuroendocrine tumors and gastric adenocarcinoma. Consequently, a surveillance esophagogastroduodenoscopy (EGD) with extensive biopsies was planned for 1–2 years later, in accordance with current guidelines. Her UC and AIH/PSC remain stable on mesalazine, Ademetionine, UDCA, with regular liver function and IBD symptom monitoring.

## Data Availability

The original contributions presented in the study are included in the article/supplementary material. Further inquiries can be directed to the corresponding authors.
